# Comparative Genome Analysis Provides Insights into the Pathogenicity of *Flavobacterium psychrophilum*

**DOI:** 10.1371/journal.pone.0152515

**Published:** 2016-04-12

**Authors:** Daniel Castillo, Rói Hammershaimb Christiansen, Inger Dalsgaard, Lone Madsen, Romilio Espejo, Mathias Middelboe

**Affiliations:** 1 Marine Biological Section, University of Copenhagen, Helsingør, Denmark; 2 National Veterinary Institute, Technical University of Denmark, Frederiksberg, Denmark; 3 Centro Nacional de Genómica y Bioinformática and Instituto de Nutrición y Tecnología de los Alimentos, Universidad de Chile, Macul, Santiago, Chile; Cornell University, UNITED STATES

## Abstract

*Flavobacterium psychrophilum* is a fish pathogen in salmonid aquaculture worldwide that causes cold water disease (CWD) and rainbow trout fry syndrome (RTFS). Comparative genome analyses of 11 *F*. *psychrophilum* isolates representing temporally and geographically distant populations were used to describe the *F*. *psychrophilum* pan-genome and to examine virulence factors, prophages, CRISPR arrays, and genomic islands present in the genomes. Analysis of the genomic DNA sequences were complemented with selected phenotypic characteristics of the strains. The pan genome analysis showed that *F*. *psychrophilum* could hold at least 3373 genes, while the core genome contained 1743 genes. On average, 67 new genes were detected for every new genome added to the analysis, indicating that *F*. *psychrophilum* possesses an open pan genome. The putative virulence factors were equally distributed among isolates, independent of geographic location, year of isolation and source of isolates. Only one prophage-related sequence was found which corresponded to the previously described prophage 6H, and appeared in 5 out of 11 isolates. CRISPR array analysis revealed two different loci with dissimilar spacer content, which only matched one sequence in the database, the temperate bacteriophage 6H. Genomic Islands (GIs) were identified in *F*. *psychrophilum* isolates 950106-1/1 and CSF 259–93, associated with toxins and antibiotic resistance. Finally, phenotypic characterization revealed a high degree of similarity among the strains with respect to biofilm formation and secretion of extracellular enzymes. Global scale dispersion of virulence factors in the genomes and the abilities for biofilm formation, hemolytic activity and secretion of extracellular enzymes among the strains suggested that *F*. *psychrophilum* isolates have a similar mode of action on adhesion, colonization and destruction of fish tissues across large spatial and temporal scales of occurrence. Overall, the genomic characterization and phenotypic properties may provide new insights to the mechanisms of pathogenicity in *F*. *psychrophilum*.

## Introduction

*Flavobacterium psychrophilum* is a yellow-pigmented, Gram-negative fish pathogen with a global distribution, causing "cold water disease" (CWD) and "rainbow trout fry syndrome" (RTFS) in freshwater aquaculture [1]. The disease results in high rates of juvenile mortality, increased predisposition to other infections and high costs of treatment with antibiotics causing significant economic losses for salmonid aquaculture worldwide [2]. Historically, the first isolation of *F*. *psychrophilum* was described in USA in 1960 [1] and isolation of *F*. *psychrophilum* as a disease-causing agent in fish farms has been reported since 1980′s in several European countries [3–5], Canada [6] and in Chile and Japan [7, 8].

Disease outbreaks can result in necrotic lesions, partial dark skin colorizing, exophthalmia, anemia, ascites, and vertebral deformities of the fish [1]. Although, pathogenesis still needs to be elucidated, proteases [9, 10], adhesins [11], secretion systems [12] and biofilm formation [13] have been proposed to play a role in pathogenicity. Treatment with antibiotics (oxolinic acid, sulfadiazine/trimethoprim, florfenicol) is still required to decrease economic losses [14], and a vaccine is currently at an early stage of development [15]. More recently, the application of specific *F*. *psychrophilum* phages to reduce the pathogen population has been suggested as an alternative method for treatment of RFTS and CWD [16, 17].

The major typing methods used to distinguish between specific *F*. *psychrophilum* strains include ribotyping, plasmid profiling and serotyping [18], multilocus sequence typing MLST [19, 20], direct genome restriction enzyme analysis (DGREA) [21] and pulsed field gel electrophoresis (PFGE) [22]. Use of these typing methods revealed a relatively low genetic diversity of *F*. *psychrophilum* on local and global scales, possibly due to efficient dispersal of the pathogen along with the massive import of fish eggs across the fish-producer countries [21, 23, 24]. From a genomics perspective, the first complete genome sequence of the virulent *F*. *psychrophilum* strain JIPO2/86 (ATCC 49511) revealed a 2,861,988-bp circular chromosome with 2432 predicted protein-coding genes. Among these predicted proteins, stress response mediators, gliding motility proteins, adhesins and putative secreted proteases are probably involved in the pathogenesis of the bacterium [12]. In addition, one Danish *F*. *psychrophilum* isolate (strain 950106-1/1) was sequenced recently, displaying the presence of novel protein secretion system, the Por secretion system (PorSS) and the secretion of extracellular enzymes at *in vitro* conditions, which was suggested to participate in adhesion, colonization and destruction of the fish tissues [13]. However, despite the economic and immediate importance of this bacterium, only a few pathogenic isolates have been sequenced and reported in the literature [12, 13, 25].

Comparative genomic analyses lend insight into structural features such as variations related to genomic rearrangements, changes in the gene repertory, identification of horizontal gene transfer elements and prophage-related sequences, and hence expose particularities on the evolution in this species [26]. These analyses have defined a conserved “core” genome shared among nearly all members of the species interspersed with “accessory” genomic elements that are present in some but absent in other strains [27]. The genome sequencing of strains belonging to the same species offers the possibility of defining their pan-genome, which comprises the core-genome and the accessory genome compartment. This strain-specific accessory genome may also be involved in critical activities of pathogenicity, drug resistance, and stress responses. While these factors may increase the adaptability of pathogens to the particular niches they inhabit, they are not imperative to the survival of the organism. Moreover, some of these genes can be acquired by horizontal gene transfer and have also been shown to be over represented in genomic islands [28]. Such pan-genome analyses have previously proved useful in identification of virulence factors in *Escherichia coli* [29] and *Streptococcus agalactiae* [30]. Thus in the present work, we have sequenced, annotated, and compared the genomic DNA sequences of six *F*. *psychrophilum* isolates from Chile and compared those with genomes of five sequenced *F*. *psychrophilum* isolates from USA, Denmark and France, allowing a comparison of isolates covering a large time scale of isolation (>65 years) and a variety of sources and geographic locations (spatial scale of isolation >12000 km). We focused specifically on characterizing the distribution of potential virulence factors, prophage content and the pan-genome in *F*. *psychrophilum*, in order to get insights into the mechanisms of pathogenicity and to establish differences in gene content that could contribute to physiological variability among the isolates.

## Materials and Methods

### Strain selection, medium composition and growth conditions

This study used 11 *F*. *psychrophilum* strains isolated from trout aquaculture in different geographic localities in Denmark, France, Chile and USA, covering a spatial scale of >12000 km and temporal scale of >60 years (S1 Table). *F*. *psychrophilum* strains MH1, PG2, VQ50, 3, 4 and 5 were isolated from Chile ([17]; this study). The strains 3, 4 and 5 were isolated from a private fish farm in Puerto Montt, Chile, with permission of the owner of the farm. Strain 950106-1/1 was isolated in Denmark [16], strains CSF259-93, FPG101 and FPG3 (ATCC 49418) were isolated in North America [6, 30] and strain JIP02/86 was isolated in France [31] (S1 Table). The isolates were stored at -80°C in broth TYES-B (tryptone 0.4%, yeast extract 0.04%, CaCl_2_ 0.05% and MgSO_4_ 0.05%) with 15% glycerol. For culturing of the *F*. *psychrophilum* strains, cells were inoculated in TYES-B medium and incubated at 15°C with agitation for 48–72 hours [16].

*F*. *psychrophilum* isolates MH1, PG2, VQ50, 3, 4 and 5 were selected for whole genome sequencing and compared with the previously sequenced strains 950106-1/1, JIP02/86, CSF 259–93, FPG3 and FPG101. Strains MH1, PG2, VQ50, 3, 4, 5, CSF 259–93, FPG3 and FPG101 were selected for phenotypic analysis (see below).

### DNA extraction

Bacterial DNA from Chilean *F*. *psychrophilum* isolates MH1, PG2, VQ50, 3, 4 and 5 were extracted from pelleted and purified isolates using the QIAamp DNA mini Kit (QIAGEN) according to manufacturer’s protocol. The amount of genomic DNA was measured using a Nanodrop2000 UV-Vis Spectrophotometer (Thermo scientific).

### Sequencing, assembly and annotation

The genomic DNA sequences of the Chilean *F*. *psychrophilum* strains MH1, PG2, VQ50, 3, 4 and 5 were obtained using Illumina HiSeq platform (BGI, China) with pair-end read sizes of 100 bp. Library construction, sequencing, and data pipelining were performed in accordance with manufacturer’s protocols. The Illumina data were assembled into contiguous sequences using Geneious software version 7.1.4, then short and low-coverage contigs were filtered out. For strains MH1, VQ50, PG2, 3, and 5 the remaining contigs were aligned using the previously sequenced *F*. *psychrophilum* strain JIPO2/86 (ATCC 49511) as reference genome (GenBank accession number: AM398681; August 2013). The DNA sequences were assembled into one scaffold with an average coverage >76x for each isolate. Gaps of unknown length (100 N's) were added adjacent to the last base, indicating a not-connection between this base and the first base. In addition, for strains MH1 and VQ50, two contigs were identified (3211 bp and 2567 bp respectively; S2 Table), which did not align with the reference genome. These contigs were localized at the end of the DNA sequences using 100 N's to represent gaps of unknown length, according to the gapped format for genome submissions specified in NCBI database. Thus, these *F*. *psychrophilum* genomes were assigned as draft genomic DNA sequences (incomplete chromosome) (S2 Table). For strain 4, the number of ambiguous bases and gaps in the genomic DNA sequence was relatively high, and the 23 contigs identified in this strain were therefore submitted to the NCBI database as a Whole Genome Shotgun (WGS) project (WGS). Finally, annotation of the contigs was achieved by the NCBI Prokaryotic Genome Automatic Annotation Pipeline (PGAAP) [32].

### Predictions of pathogenic and resistance islands, virulence factors and prophages

We used PAI finder [33] and PIPS [34] to predict the putative genomic islands (GIs) and antimicrobial resistance islands (REIs) in *F*. *psychrophilum* isolates. In addition, a genome comparison carried out by MAUVE [35] was used to identify GIs which were not recognized by PAI finder or PIPS. The virulence database MvirDB [36] was used to predict putative virulence factors. All predicted genes of the 11 *F*. *psychrophilum* isolates were searched against the MvirDB by blastp with loose criteria (E-value≥1e-5; identity≥35%; coverage≥80%). Also, virulencefinder 1.2 [37] was used to screen putative virulence factors using selected database from *Escherichia coli*, *Enterococcus* and *Streptococcus aureus*. Prophage-related sequences were identified by running bacterial genomes in phage_finder v2.1 [38] and PHAST [39].

### CRISPR array detection

The putative CRISPR loci for 11 isolates were identified with CRISPRfinder [40]. Spacer sequences were aligned to the whole genome sequenced *F*. *psychrophilum* bacteriophages FpV4 (90 kb), FpV9 (48 kb), FpV21 (90 kb) and 6H [41] using ClustalW algorithm in Geneious 7.1.4 program [42].

### Pan genome analysis

In order to predict the possible genomic changes in *F*. *psychrophilum*, the bioinformatics program EDGAR [43] was used to predict pan genome of all 11 *F*. *psychrophilum* isolates and calculate the pan-genome (gene repertoire), accessory genome (specific genes, only found in one genome) and core genome (common genes, mutually conserved). Pan-genome development of was calculated by iterative pairwise comparison of a set of genomes. Using the metacontig function of EDGAR, we also defined custom groups of *F*. *psychrophilum* genomes for which the core genome or the pan genome have been stored as virtual contigs [43].

### Phylogenetic analysis

To determine the phylogenetic relationship among *F*. *psychrophilum* isolates based on genomic data, we selected a set of orthologous genes shared by all 11 isolates (1426 genes present in a single copy, paralogs not included) and *F*. *columnare* ATCC 49512 (outgroup to root the three) using OrthoMCL with an e-value cut off 10^−10^ [44]. The set of 1426 single core genes were first aligned at amino acid level using Clustal W version 2.0 [45], then back translated to DNA sequences using PAL2NAL [46]. The alignment of all orthologous genes was concatenated using FASconCAT [47]. Gene tree was constructed using PhyML [48].

### Biofilm formation

Biofilm formation was quantified using the standard assay with crystal violet staining of biofilm and subsequent measurement of the optical density at 595 nm as previously described for *F*. *psychrophilum* [12]. The ten isolates were grown in half strength TYES-B to mid-exponential phase (10^8^ cells/ml). *F*. *psychrophilum* phage resistant isolate V1-20 was used as a negative control [13]. The cultures were diluted 1/100 in TYES-B and then 1 ml of each dilution was inoculated in quadruplicate into polystyrene tubes (Becton Dickson, Falcon), which were incubated statically at 15°C for 5 days. Following this incubation, the supernatants were discarded, and the tubes were washed seven times with 1 ml of sterile distilled water. Then 1 ml of a 1% (wt/vol) crystal violet solution (Sigma-Aldrich) was added to each polystyrene tube containing the cells. After 45 min, the crystal violet solution was removed, the wells were washed seven times with 1 ml of sterile distilled water to remove the unbound dye, and then 1 ml of 96% (vol/vol) ethanol was added to release attached cells from the tube surface. Biofilm formation was then quantified by measuring the optical density at 595 nm. Experiments were repeated in four independent assays.

### Motility

The ability of the ten isolates to move by gliding were examined by inspection of motility on glass microslides [13], by using the hanging drop technique [49], as well as by using agar plates as surfaces for the gliding motility [50]. Briefly, all the isolates were grown in TYES-B for 48–72 h at 15°C. Glass capillary microslides (Camlab) were introduced in the bacterial cultures and motility was observed using phase contrast microscopy (Olympus BX61). In an additional approach, one drop of liquid culture of each strain was deposited onto a cover slip, which was turned upside down and placed on tiny stands on a glass slide. Bacterial motility was observed through the cover slip. Finally, aliquots of 5 μL bacterial culture were spread on plates with 2x, 1x, 0.5x, and 0.1x and 0.01x diluted TYES-B agar (1.1%). After 72 h incubation, the colony diameters were measured as an estimate of dispersal rate by gliding motiliy. Experiments were repeated in four independent assays.

### Secretion of extracellular proteins

The activities of the extracellular enzymes proteolysins, and gelatinase were measured in each strain as previously described for *F*. *psychrophilum*, including the phage resistant isolate V1-20 [13]. 50 μl aliquots of bacteria-free supernatant (0.45-μm-filtration) from three replicates of a 4–5 day liquid culture of each isolate with cell densities were added to holes punched into agar plates. For total proteolytic activity, cells were added to agar plates containing 2% skim milk and plates were incubated at 15°C overnight. Gelatinase activity was determined using 2% gelatine plates and incubated at 4°C. Experiments were repeated in four independent assays.

### Hemolytic activity

Bacterial hemolytic activity was assessed using the microplate hemolysis assay described by Högfors-Rönnholm and Wiklund [51]. Blood was collected in an equal volume of Alsever’s solution (Sigma Aldrich) by caudal venipuncture of rainbow trout (about 800 g). Erythrocytes were centrifuged (1000 x g, 5 min, 4°C), washed three times with phosphate buffered saline (PBS, pH 7.2). For the assay, washed and packed erythrocytes were suspended to 5% (v/v) in PBS. An equal amount (30 μl) of erythrocyte and bacterial suspensions (including the phage resistant strain V1-20 as a negative control [13]) were mixed in triplicates into a U-well microtiter plate (Greiner bio-one) and incubated for 24 h at 10°C and 400 rpm rotation. Following incubation, 150 μl 0.5% NaCl was added to the wells and the plate was centrifuged (1000 x g, 5 min, 4°C). The supernatants were transferred to a F-well microtiter plate (Greiner Bio-one) and the absorbance was measured at 540 nm (A). A negative control (background, A^background^) with only 0.5% NaCl and erythrocytes and a positive control (total hemolysis, A^100%^) with distilled water and erythrocytes were included in triplicates on each plate. The hemolytic activity was calculated according to: Hemolytic activity = (A-A^background^)/ (A^100%^-A^background^). Experiments were repeated in four independent assays.

### Statistical analysis

A Student’s t-test (GraphPad Prism 4 software) was used to analyze the statistical significance of the observed differences in activities among the strains. P-values of <0.01 were defined as statistically significant.

### Accession numbers

Accession numbers for *F*. *psychrophilum* strains are listed in the S1 Table. Bacteriophages 6H, FpV4, Fpv9 and Fpv21 have been assigned GenBank accession numbers: KC959568, KT876724, KT876725 and KT876726 respectively.

## Results

### General features and architecture of the *F*. *psychrophilum* genomes

The DNA sequences were obtained for *F*. *psychrophilum* isolates MH1, PG2 and VQ50, which are responsible for salmon and rainbow trout infections in Chile (S1 Table) [17]. The *F*. *psycrhophilum* strains 3, 4 and 5 were isolated from water samples collected from fish farms in Chile. These six DNA sequences were analyzed together with the genomes of isolates 950106-1/1, JIP02/86, CSF 259–93, FPG3 and FPG101 already available in the databases (S1 Table). The 11 *F*. *psychrophilum* isolates varied in size from 2.71 and 2.86 (Mb) with a GC content of 32.4–32.7%. One plasmid was present in the isolate JIP02/86 (Table 1). Analysis of annotated contigs revealed a relatively similar ORF number among all the isolates (2406–2569 ORFs).

**Table 1:** Summary of genome sequence projects of *F*. *psychrophilum* isolates.

Isolate	Geographical Origin	Size (Mb)	Genes	CDS	Pseudogenes	%GC	rRNA operons	tRNA	Plasmid	Reference
FPG3 (ATCC 49418)	USA	2.71	2406	2310	28	32.7	6	49	None	[52]
4	Chile	2.71	2494	2344	49	32.4	6	36	None	This study
CSF 259–93	USA	2.90	2569	2467	34	32.5	6	49	None	[25]
FPG101	Canada	2.83	2516	2424	24	32.6	6	49	None	[6]
3	Chile	2.80	2482	2390	24	32.6	6	49	None	This study
5	Chile	2.84	2539	2447	24	32.5	6	49	None	This study
PG2	Chile	2.85	2541	2451	22	32.5	6	49	None	[17]
950106-1/1	Denmark	2.74	2459	2396	21	32.4	1	49	None	[13]
JIP02/86 (ATCC 49511)	France	2.86	2556	2446	43	32.5	6	49	pCP1	[12]
MH1	Chile	2.84	2541	2447	26	32.6	6	49	None	[17]
VQ50	Chile	2.80	2483	2390	25	32.5	6	49	None	[17]

### Pan-genome analysis

**Fig 1:**
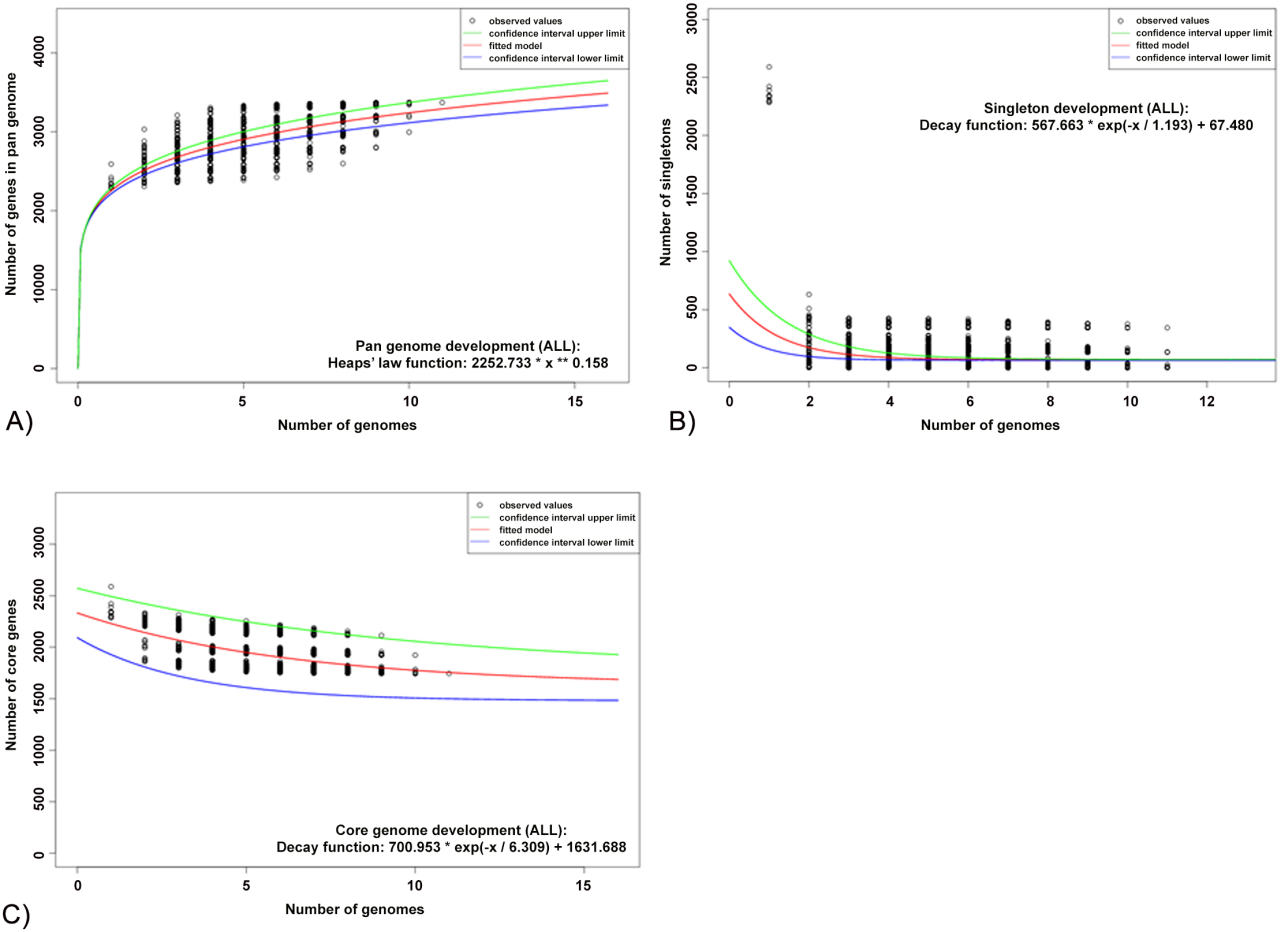
*F*. *psychrophilum* pan, core and accessory genome evolution according to the number of sequenced genomes. A) Total number of genes (pan-genome) for a given number of genomes sequentially added. The pan genome analysis is shown for increasing values for every *F*. *psychrophilum* genome sequenced based on a power law fit model. B) Number of shared genes (core genome) as a function of the number of genomes sequentially added. The exponential decay model based on the median value for the conserved core genes displays that the core genome had a minimum of 1743 genes in *F*. *psychrophilum*. C) Number of unique genes (accessory genome) for a given number of genomes sequentially added. Decreasing number of accessory genes per genome was observed with increased of genomes examined. The graphic shows the exponential decay model based on the median value for the accessory genes when increasing numbers of genes were analyzed. An average of 67 new genes could be detected in the *F*. *psychrophilum* pan genome for each new genome added. For all the plots the upper (green) and lower (blue) lines indicate the first (25th percentile of the data) and third (75th percentile) quartiles respectively. The central (red) line refers the sample median (50th percentile) of random input order of the *F*. *psychrophilum* genomes.

Examination of the *F*. *psychrophilum* pan-genome indicated that the gene repertoire increased with sequential addition of each new genome, and continued to increase for all additions (Fig 1A). The number of new genes supplied by a novel genome was 201 for the second genome added and 76 for the last, and the model predicted that the gene repertoire of *F*. *psychrophilum* could hold at least 3373 genes. In order to determine if the *F*. *psychrophilum* pan-genome was open, the accessory genes were calculated from incorporation of a new genome sequence every time. We therefore applied the exponential decay model to identify unique genes using the median value. The model estimated that 67 ± 3 new genes could be revealed for every new *F*. *psychrophilum* sequence added to the analysis (Fig 1B). This relatively high rate of increase in the pan-genome size with addition of new genomes suggested that *F*. *psychrophilum* possesses an open pan-genome.

In contrast to the pan-genome, an examination of the *F*. *psychrophilum* core genome showed that the number of shared genes decreased with the addition of each new genome, as expected (Fig 1C). The *F*. *psychrophilum* average gene content is 2375 ± 54 genes and the core genome was estimated to contain 1743 ± 51 genes, corresponding to 73% of the genome which would remain relatively constant even if additional isolates genomes were included.

### Virulence factors

For examination of the possible molecular basis for virulence phenotypes, we compared the *F*. *psychrophilum* genomes with respect to virulence gene content to provide additional insights into the biology and evolution of this fish pathogen. Using databases from MvirDB and virulencefinder did not detect any virulence factors. Previous *F*. *psychrophilum* genome studies have, however, detected putative gene candidates participating in virulence, such as genes encoding proteases and adhesion [12], transport [52], motility and a specific secretion system [53]. These 44 putative virulence factors among 11 *F*. *psychophilum* genomes showed a remarkably similar distribution in the strains independent of geographic locality, year of isolation or source of isolates, except for a putative hemolysin D transporter (*hlyD*), which was found only in the isolates CSF 259–93, FPG101 and 4 (S3 Table).

### Strain-specific genomic islands

**Fig 2:**
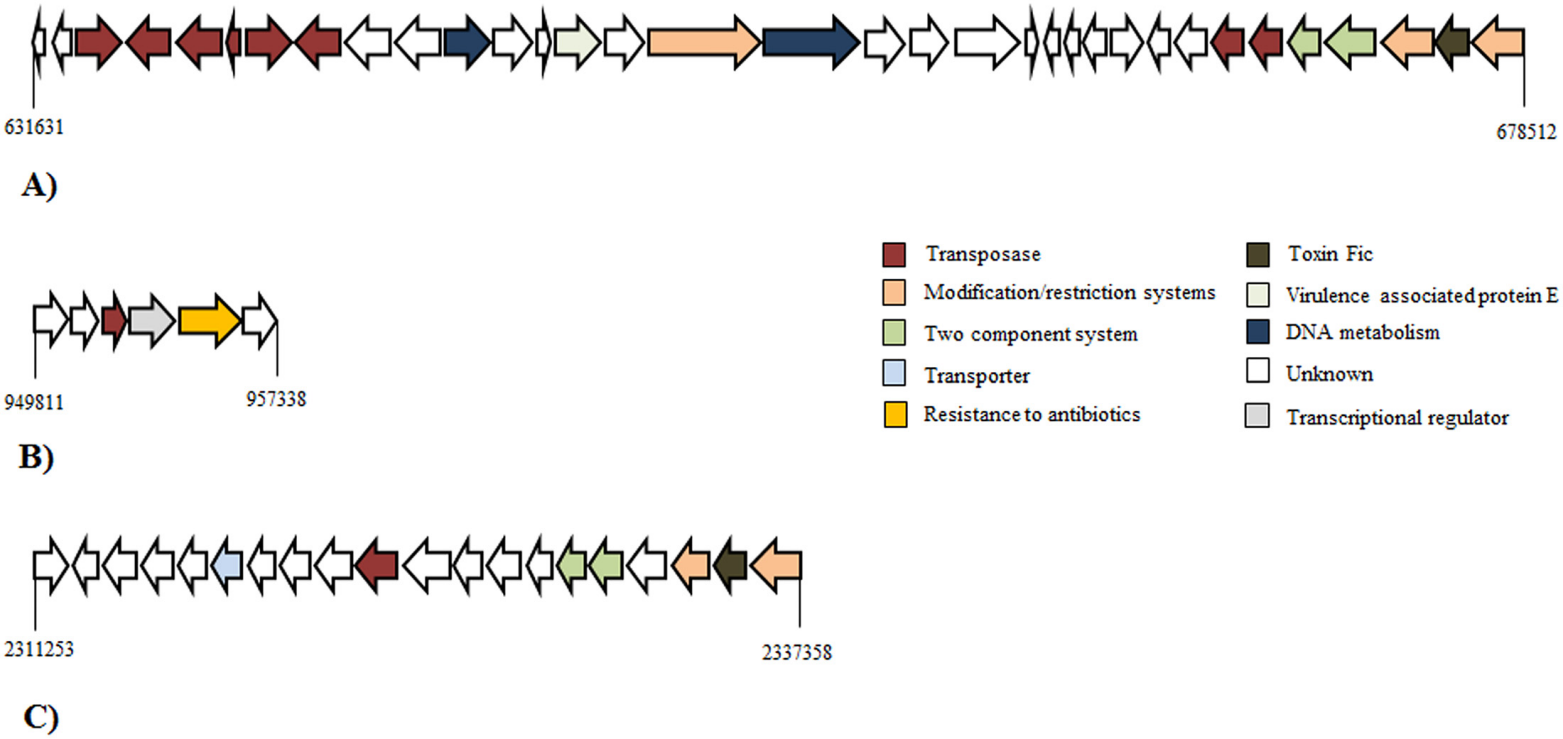
Schematic representation of Genomic islands (GI) present in the *F*. *psychrophilum* isolates. A) GI found in *F*. *psychrophilum* CSF 259–93 associated with toxin Fic and virulence factor E family. B) GI found in *F*. *psychrophilum* CSF 259–93 associated with tetracycline resistance. C) GI found in *F*. *psychrophilum* 950106-1/1 associated with toxin Fic. The colors were assigned according to the possible role of each ORF.

Genomic islands (GI) are defined as cluster genes in prokaryotic genomes of probable horizontal origin and commonly encoding mobility related genes, and genes involved in virulence and drug resistance [54]. We examined the genomic distribution of GI in the 11 *F*. *psychrophilum* isolates in order to find unique acquired regions associated with virulence genes. Only a subset of strains (950106-1/1, CSF 259–93) displayed GIs detected by bioinformatics tools (Fig 2; S4 and S5 Tables). Functions assigned to GI genes ranged from transposases, modification and restriction systems, resistance to antibiotics, virulence factors, toxins, DNA metabolism and unknown functions. Unfortunately, parts of these genes with unknown functions have been poorly characterized, and their biological significance was difficult to infer based on available annotations. The largest GI found was present in the isolate CSF 259–93 (46.8 kb), and contained genes encoding toxin Fic (WP_034100128.1), virulence associated protein E (VapE), type II restriction endonucleases and one two- component system associated with multiple tranposases (Fig 2A). Moreover, another GI in the same isolate was associated with an antibiotic resistance (Fig 2B). Finally, the Danish isolate 950106-1/1 contained a GI of 28.5 kb, which encoded a toxin Fic (Fig 2C).

Other putative GIs (arbitrarily >3 ORFs; 388 ORFs total) were detected as non-aligned regions using Mauve alignment. Of these genes, 269 belonged to *F*. *psychrophilum* isolate 4 (69%). For example, a unique region of 9.4 kb with genes encoding transport, multi-drug resistance and heat shock proteins was found in this isolate. Part of these proteins showed amino acid similarity to homologous genes of *Flavobacterium sp*, *Chryseobacterium sp*, *Riemerella colombina* and other bacteria belonging to the phylum *Bacteriodetes* (data not shown). The predicted functions assigned each GIs are showed in the supplementary information (S5 Table).

### Prophages

We examined phage-like elements in the genomes in order to determine the prophage carriage in *F*. *psychrophilum*. Except from the presence of the previously characterized 45-kb prophage 6H [41], which was found in the *F*. *psychrophilum* isolates 950106-1/1, JIP02/86, MH1, PG2 and 5, the data did not reveal phage-related regions in genomes (data not shown).

### Identification of CRISPR loci in *F*. *psychrophilum* isolates

**Fig 3:**
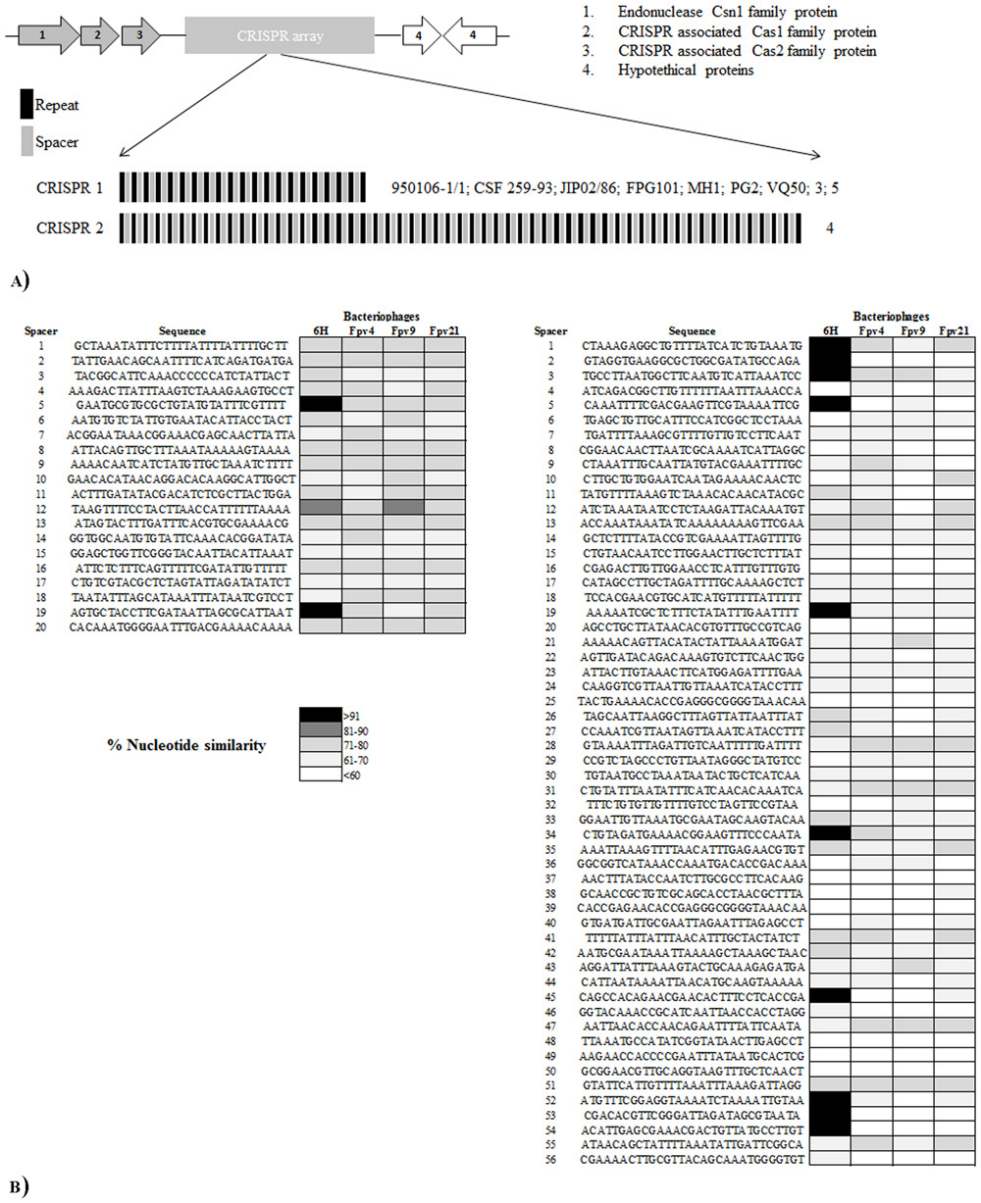
*F*. *psychrophilum* isolates CRISPRs overview. A) Graphic representation of *cas* genes, spacer and repeats of CRISPR1 and CRISPR2 in the *F*. *psychrophilum* isolates. B) Nucleotide comparison of spacers from CRISPR1 (left) and CRISPR2 (right) against genome sequences from bacteriophages 6H, Fpv4, Fpv9 and Fpv21.

In order to characterize the CRISPR/Cas system within *F*. *psychrophilum* isolates, we examined the 11 DNA sequences for CRISPR arrays. The results showed an identical CRISPR array linked to three putative cas proteins, encoded by genes- *csn1*, *cas1* and *cas2*, that exhibited 20 different spacers of 29–30 bp and 21 direct repeats of 46 bp for *F*. *psychrophilum* isolates 950106-1/1, CSF 259–93, JIP02/86, FPG101, MH1, PG2, VQ50, 3 and 5 (nominated CRISPR1) (Fig 3A). *F*. *psychrophilum* strain 4, on the other hand, showed a CRISPR array associated to cas proteins with 57 direct repeats and 56 spacers (nominated CRISPR 2; Contig 4) (Fig 3A). No CRISPR arrays were found in strain FPG3. We examined the nucleotide similarity of *F*. *psychrophilum* spacers from all CRISPRs with sequences of 4 known *F*. *psychrophilum* bacteriophages (6H, FpV4, FpV9 and FpV21) and other sequences of extrachromosomal origin in the NCBI database. The results for CRISPR 1 revealed that 19 out 21 spacers did not significantly match (<80% similarity) with genomic sequences from *F*. *psychrophilum* bacteriophages Fpv4, Fpv9, Fpv21 or with sequences in the NCBI database. The two exceptions were the spacers 5 and 19, which showed 100% and 97% similarity, respectively with sequences of the temperate bacteriophage 6H [41] (Fig 3B). In the same way, spacers from CRISPR 2 showed no significant match with genomes from bacteriophages FpV4,-9, -21, or any sequence from database, but 10 spacers displayed 100% similarity with sequences from bacteriophage 6H (Fig 3B). The ORFs targeting the bacteriophage 6H are shown in the additional information (S6 Table).

### Phylogeny

**Fig 4:**
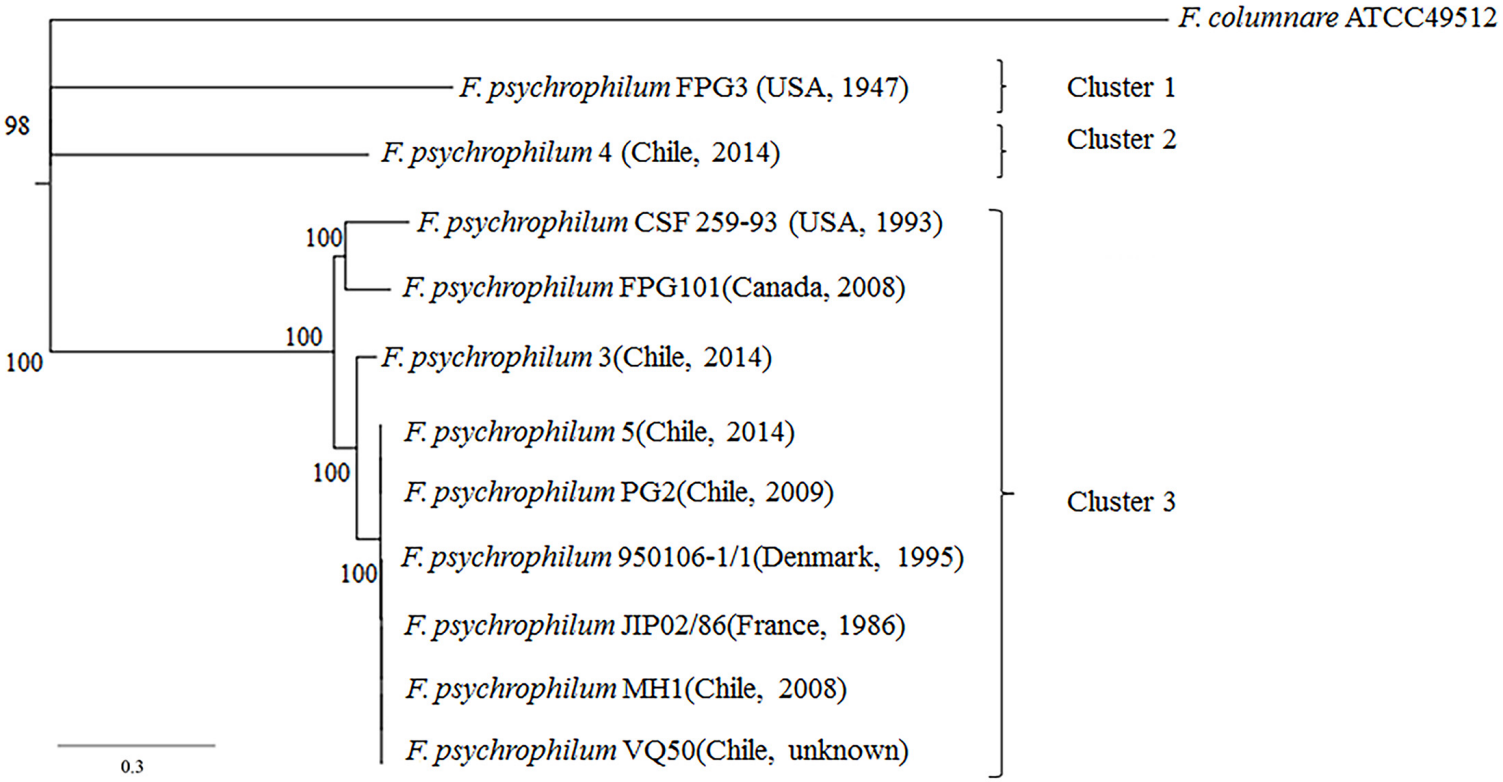
Phylogenetic tree inferred from concatenated genes. Maximum likelihood tree obtained from a concatenated nucleotide sequence alignment of the orthologous core genes for the 11 *F*. *psychrophilum* isolates and *F*.*columnare* ATCC49512 (outgroup). The horizontal bar at the base of the figure represents 0.3 substitutions per nucleotide site. The percentages of genes that support the branches of the tree are indicated. Geographic location and isolation year of *F*. *psychrophilum* isolates were added to facilitate comparison.

We determined the overall phylogenetic relationship from a concatenated alignment of 1426 single-copy orthologs shared by all 11 *F*. *psychrophilum* isolates, rooted by *F*. *columnare* ATCC49512 (outgroup) (Fig 4). Three different clusters can be visualized: Cluster 1 including the American isolate FPG3. Cluster 2 was formed by the Chilean isolate 4. Finally, cluster 3 which contained the North American isolates CSF 259–93 and FPG101, French isolate JIP02/86, Danish isolate 950106-1/1 and the Chilean isolates MH1, VQ50, PG2, 5 and 3, which showed a temporal scale of isolation >28 years (Fig 4). The phylogenetic tree indicated that 5 out of the 6 Chilean *F*. *psychrophilum* isolates (MH1, VQ50, PG2, 4 and 3) had a common ancestor. The American and Chilean *F*. *psychrophilum* isolates FPG3 and 4, respectively, displayed the most distant linages in our phylogenetic tree with 98% support (Fig 4).

### Phenotypic properties

Ten *F*. *psychrophilum* isolates were phenotypically analyzed for gliding motility, biofilm formation and secretion of extracellular enzymes (S1 Fig). None of the strains showed motile behavior in any of the conditions tested (data not shown), and no significant differences (p>0.01) in biofilm formation, hemolytic activity, gelatinase activity and total protease activity was observed among the strains (S1 Fig).

## Discussion

### The *F*. *psychrophilum* pan-genome

Regression analysis suggested that the *F*. *psychrophilum* pan-genome can be categorized as an open pan-genome because the gene repertoire could hold at least 3373 genes (Fig 1A) according to the model, and as many new genes (67 ±3) could be identified for every new genome added to the analysis (Fig 1C). In contrast to that, the size of the core genome decreased with the addition of each new sequenced isolate, representing on average 73% of the pan-genome (Fig 1B). Thus, these results demonstrated that the 11 *F*. *psychrophilum* isolates only represented a subset of the genetic diversity in this species and suggested that the *F*. *psychrophilum* pan-genome is still evolving by gene acquisition and diversification. Similar results have been described for *Erwinia amylovora* [55], *Streptococcus agalactiae* [30] and *Propionibacterium acnes* [56], whereas analysis of *Bacillus anthracis* suggested a closed pan-genome in which the diversity was covered with only four isolates [57].

The indication of an open pan-genome in *F*. *psychrophilum* is somewhat surprising given the relatively restricted ecological niche as a fish pathogen. However, little is known about the ecology of *F*. *psychrophilum* and our study support previous studies demonstrating a high recombination rate among *F*. *psychrophilum*, likely mediated by mobile elements [21, 23, 58], which can contribute to accessory gene repertory in *F*. *psychrophilum*.

### Virulence factors and prophage identification in *F*. *psychrophilum*

Virulence factors of pathogenic bacteria play an important role in colonizing various niches through infection of their host and adaptations to new environmental conditions [59]. All *F*. *psychrophilum* isolates showed similar content and distribution of adhesion and metalloproteases proteins, which could play important roles in attachment and destruction of host tissues respectively [9, 60]. Moreover, the gliding motility and Por secretion system (PorSS) proteins were detected in all the isolates (S3 Table). These proteins are widespread among members of the phylum *Bacteriodetes* [61] and have been associated with the transport and translocation of virulence factors in the pathogens *F*. *johnsoniae* and *Porphyromonas gingivalis* [53]. Besides, genome analysis focusing on resistance mechanisms in phage-resistant *F*. *psychrophilum* clones displayed a link between mutations in the proteins of PorSS system and decreased of virulence properties at *in vitro*, suggesting an important role in virulence for this bacterium [13].

The presence of prophage 6H in 5 of the genomes confirmed a recent study which showed a wide spread and global distribution of this prophage in the genomes of *F*. *psychrophilum* isolates [41]. Prophage 6H has been associated with putative virulence factors; however, it is not known to what extent prophage genes are participating in bacterial infection. Interestingly, no other prophage-like sequences were detected in *F*. *psychrophilum* genomes (data not shown). Similar findings have been described for *F*. *banchophilum*, *F*. *columnare* and *F*. *johnsoniae*, where genome sequences are lack of prophage-like elements [62]. Moreover, a large number of lytic phages (>20) have been isolated against *F*. *psychrophilum* [16], and the absence of these phages as prophages in the sequenced strains suggested that their integration through a lysogenic life cycle is rare. On the other hand, the rapid loss of prophage 6H after exposure to phages in *F*. *psyhcrophilum* strain 950106-1/1 [21] also indicates a dynamic and temporary occurrence of prophages due to the integration/excision to and from *F*. *psychrophium* genomes. However, the dynamics of prophages is not resolved in this study.

### Genomic islands and strain-specific genes in *F*. *psychrophilum*

A total of 3 specific GIs were detected in the 11 *F*. *psychrophilum* isolates using PAIfinder and PIPS tools (Fig 2; S4 Table). *F*. *psychrophilum* isolates CSF 259–93 and 950106-1/1 showed GIs of 46.8 kb and 25.8 kb respectively, associated to tranposases, modification-restriction systems, virulence associated protein E (VapE) and toxin Fic (Fig 2A and 2C). Fic proteins have emerged as a new class effector that interferes with host cell signaling pathways, affecting indirectly cytoskeletal dynamics [63]. Although a similar gene has been identified previously in *F*. *psychrophilum* strains, they were not associated to mobile elements in these strains [6, 12]. Virulence associated protein E (VapE) was originally identified and associated with virulence in *Dichelobacter nodosus* [64] and *Staphylococcus aureus* [65], but the mechanism by which this protein affects virulence has not been determined. Finally, isolate CSF 259–93 also presented a small GI of 7.5 kb associated to tetracycline resistance gene (Fig 2B), confirming previous observations of resistance to this specific antibiotic [66]. In the current analysis, we defined arbitrarily GIs as genomic regions containing three or more ORFs, which were specific for a subset of the *F*. *psychrophilum* isolates. A total of 388 strain-specific ORFs were detected among the isolates 950106-1/1; CSF 259–93, FPG3 and 4 (S5 Table). Although genes associated with transposases, DNA metabolism, transport and hydrolases were found among these, hypothetical genes or unknown genes represented the vast majority of these unique regions, and the similarity of these new genes between bacteria belonging to the phylum *Bacteriodetes* suggests a possible acquisition though horizontal transfer.

### CRISPR identification in *F*. *psychrophilum*

Two different CRISPR systems were found among *F*. *psychrophilum* isolates used in this study (Fig 3). Previous studies have determined the presence of CRISPR 1 in the *F*. *psychrophilum* isolates 950106-1/1 and JIP02/86 [12], but the current work demonstrated the presence of this specific CRISPR system also in the isolates CSF 259–93, FPG101, MH1, PG2 VQ50, 3 and 5 (Fig 3A). Interestingly, *F*. *psychrophilum* isolate 4 contained a different CRISPR array (CRISPR 2) with 56 spacers and 57 direct repeats (Fig 3A). The nucleotide similarity of spacers against a bacteriophage collection showed that both CRISPR systems contained spacers which matched with bacteriophage 6H, but with variable numbers of 6H specific spacers (Fig 4B; S6 Table). These findings support the hypothesis that bacteriophage 6H belongs to an abundant group of cosmopolitan temperate phages which have lysogenized a large fraction of the global *F*. *psychrophilum* community [41]. The stability of CRISPR1 across the large temporal and geographical distances between the isolation of the CRISPR1-containing strains suggested that this system is not an active and dynamic phage defense mechanism in *F*. *psychrophilum*. Alternative roles for CRISPR systems have been described in pathogenic bacteria [67]. For example, when *P*. *aeruginosa* is lysogenized by a specific bacteriophage, the CRISPR/Cas system interacts with a particular gene in the chromosomally integrated prophage to inhibit the creation of biofilms [68]. We have previously suggested that prophage 6H could play a role in the virulence properties of *F*. *psychrophilum* [41], but we do not know whether CRISPR in *F*. *psychrophilum* contributes to the regulation of prophage 6H genes.

### Phylogenetic analyses

The phylogenetic analyses based on a concatenated alignment of 1426 single-copy orthologs shared for all the *F*. *psychrophilum* isolates revealed close relationships among the diverse isolates of *F*. *psychrophilum*, across large temporal and geographic scales of isolation (Fig 4). For example, Chilean *F*. *psychrophilum* isolates PG2, MH1, VQ50, 3 and 5 showed less diversity and grouped together with Danish, French and North American isolates 950106-1/1, JIP02/86, CSF 259–93 and FPG101 respectively (cluster 3) (Fig 4). In the same way, Chilean isolate 4 and American isolate FPG3 clustered in a distant lineages (cluster 1 and 2). These observations are consistent with studies using multi locus sequence typing (MLST), where the Chilean *F*. *psychrophilum* isolates were closely related to the genotypes most prevalent in European and North American fish farms [24], probably due to massive trade of fish eggs across the fish-producer countries [21, 23, 24]. Further, the high similarities in orthologous genes among strains isolated in France, Denmark and Chile over a 28 year period (Cluster 3, Fig 4) suggest that these core genes are highly conserved and that the strain specific genomic differences in *F*. *psychrophilum* are mainly due to gain and loss of mobile genetic elements. More extensive genomic samplings are required to allow a more detailed phylogenetic analysis among *F*. *psychrophilum*, and to establish the relationships of the core genome across the genus level.

### Phenotypic traits in *F*. *psychrophilum*

Phenotypic characterization showed similar properties of 10 *F*. *psychrophilum* isolates with respect to biofilm formation, hemolytic activity and secretion of extracellular enzymes (S1 Fig). These results are in agreement with our observations of a homogenous distribution of adhesion and metalloproteases proteins in all *F*. *psychrophilum* isolates (S3 Table). Moreover, components of Por secretion system (PorSS) were found in all the isolates (S3 Table), suggesting that *F*. *psychrophilum* is proficient in protein secretion through the PorSS, as is proposed for *F*. *branchiophilum* [62].

Despite the identification of genes involved in motility (*gld*) (S3 Table) no motility was observed in any of the isolates in the conditions tested (data not shown). However, we cannot rule out that *F*. *psycrhophilum* may be motile, but experimental conditions used here failed to simulate natural conditions where gliding motility proteins could be expressed.

### Conclusion and future directions

We report here the DNA sequences of six Chilean *F*. *psychrophilum* isolates (MH1, PG2, VQ50, 3, 4 and 5) and compared them with 5 previous genome sequences from North America, Denmark and France. Our genome analysis clearly showed that Pan-genome is open in *F*. *psychrophilum* (Fig 1) and many more sequences are required to cover the diversity of this bacterial species. The identification and characterization of core and accessory genes is essential for understanding the basic metabolism in *F*. *psychrophilum*, for providing a better insight on virulence properties, and choice of antibiotic treatment, and for further drug and vaccine development, as have been proposed for universal group B of *Streptococcus* [69]. Future studies about the estimation of the Pan genome in the genus *Flavobacterium* could provide insight into species differentiation and enhance our understanding of evolution within the *F*. *psychrophilum* cluster (Fig 4).

The distribution of putative indicators of virulence, previously identified in the *F*. *psychrophilum* isolates JIP02/86 and FPG3, was similar in all the geographic distant isolates (S3 Table). This large scale dispersion of virulence factors may explain the similar ability to form biofilm and to secrete extracellular enzymes among the strains (S1 Fig). Consequently, we can speculate that *F*. *psychrophilum* isolates have a similar mode of action on adhesion, colonization and destruction of fish tissues across large spatial and temporal scales of occurrence. However, the presence of an extra Fic toxin in genomic islands identified in the strains CSF 259–93 and 950106-1/1 (Fig 2) could indicate that those isolates differed on some steps of infection, probably causing a major effect of destabilization of the cytoskeleton on host cells. In the same way, several strain-specific genetic elements were also found within the genomes of isolates FPG3 and 4 and could be participating in the infection of the host (S5 Table).

Altogether, our results allowed us to gain a better understanding of *F*. *psychrophilum* pathogenicity and diversity. Defining the pan genome, distribution of virulence factors, mobile elements and phylogenetic relationship and how this genetic information contributes to the phenotype in *F*. *psychrophilum*, may help developing diagnostic tools and strategies for the control of this pathogen.

## Supporting Information

S1 Fig Phenotypic characterization of *F*. *psychrophilum* isolates. A) Biofilm formation. B) Hemolytic activity. C) Gelatinase activity on gelatin plates. D) Total protease activity on skim milk plates.
(DOCX)

S1 Table Characteristics of *F*. *psychrophilum* isolates analyzed in this study. (DOCX)

S2 Table Genome characteristics of Chilean *F*. *psychrophilum* isolates. (DOCX)

S3 Table Distribution of putative virulence factors across *F*. *psychrophilum* isolates. (DOCX)

S4 Table Unique genome regions identified in *F*. *psychrophilum* isolates using PAI finder. (DOCX)

S5 Table Unique genome regions identified in *F*. *psychrophilum* isolates using MAUVE program. (DOCX)

S6 Table Target genes on bacteriophage 6H by CRISPR spacers. (DOCX)
