# Supplementary material for: Comparative Genome Analysis Provides Insights into the Pathogenicity of Flavobacterium psychrophilum
Source: PLoS One. 2016 Apr 12;11(4):e0152515. doi: 10.1371/journal.pone.0152515 (PMC4829187; doi:10.1371/journal.pone.0152515)
Supplement: S1 Fig — A) Biofilm formation. B) Hemolytic activity. C) Gelatinase activity on gelatin plates. D) Total protease activity on skim milk plates. (DOCX) [file pone.0152515.s001.docx]

**Phenotypic characterization of *F. psychrophilum* isolates**

In order to examine the phenotypic characteristics of *F. psychrophilum* isolates, we evaluated the biofilm formation and the levels of extracellular enzymes in the supernatants of bacterial cultures. These analyses indicated that all the isolates exhibited equally biofilm formation, hemolytic activity, gelatinase activity and total protease activity. *F. psychrophilum* phage resistant strain V1-20 was added to the analysis as a negative control.


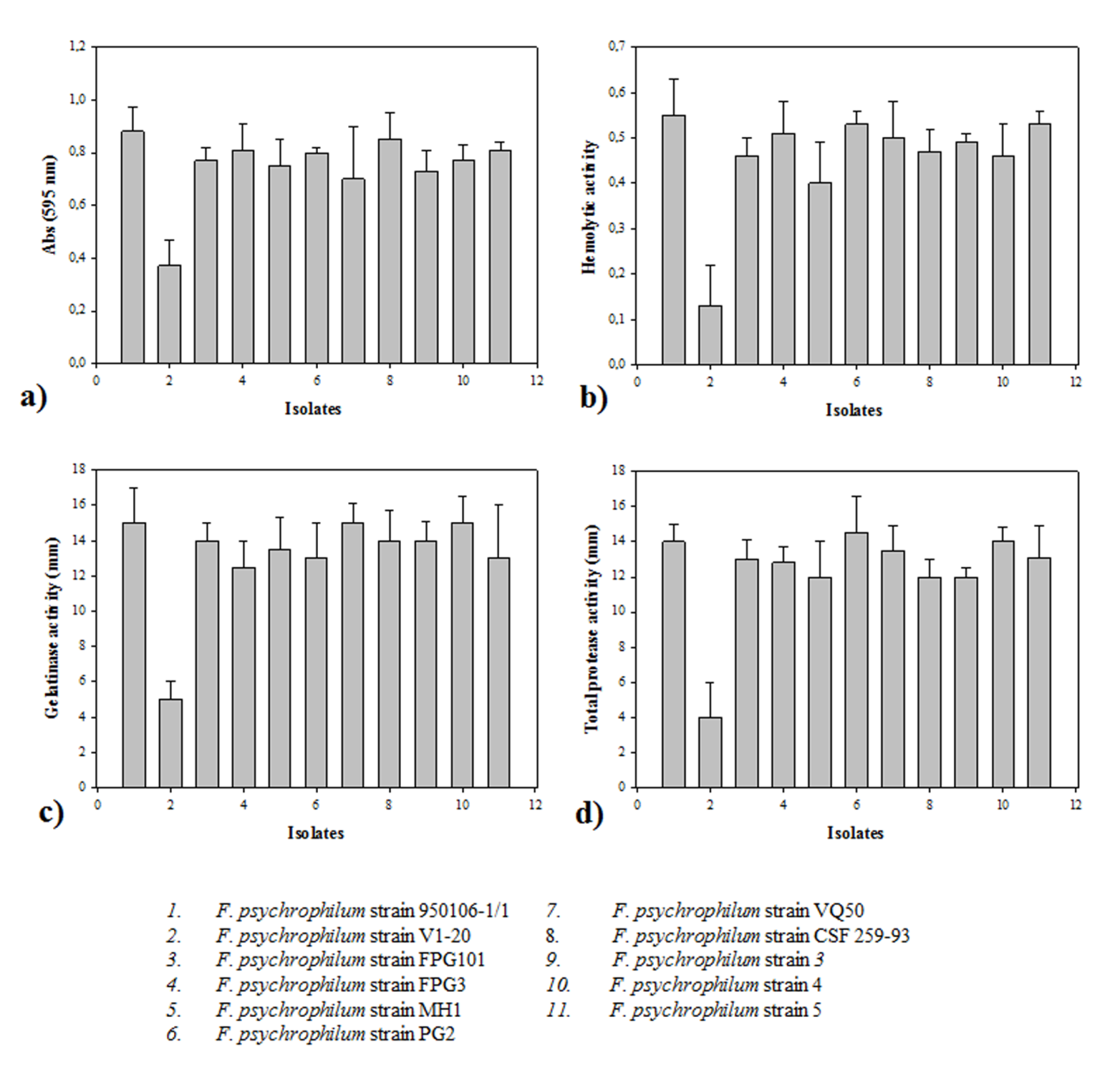


**Fig 1S. Phenotypic characterization of *F. psychrophilum* isolates.** A) Biofilm formation. B) Hemolytic activity. C) Gelatinase activity on gelatin plates. D) Total protease activity on skim milk plates.
