# Supplementary material for: Comparative Genome Analysis Provides Insights into the Pathogenicity of Flavobacterium psychrophilum
Source: PLoS One. 2016 Apr 12;11(4):e0152515. doi: 10.1371/journal.pone.0152515 (PMC4829187; doi:10.1371/journal.pone.0152515)
Supplement: S1 Table — (DOCX) [file pone.0152515.s002.docx]

**Genomics characteristics of *Flavobacterium psychrophilum* isolates**

The Table 1S shows additional information about the geographic locality, source, year of isolation and sequencing status of all the *F. psychrophilum* isolates. Accession numbers are also added.

Table 1S. Characteristics of *F. psychrophilum* isolates analyzed in this study

| Isolate | Geographical origin | Source | Year of isolation | Sequencing status | Accession number |
| --- | --- | --- | --- | --- | --- |
| 950106-1/1 | Denmark | Rainbow trout | 1995 | Complete | CP008902 |
| JIP02/86 | France | Rainbow trout | 1986 | Complete | AM39861 |
| MH1 | Chile | Atlantic salmon | 2008 | Draft | CP010275 |
| PG2 | Chile | Rainbow trout | 2009 | Draft | CP010276 |
| VQ50 | Chile | Rainbow trout | Unknown | Draft | CP010277 |
| 3 | Chile | Water sample | 2014 | Draft | CP010278 |
| 4 | Chile | Water sample | 2014 | Draft | LRTL00000000 |
| 5 | Chile | Water sample | 2014 | Draft | CP010274 |
| CSF 259-93 | USA | Rainbow trout | 1993 | Complete | CP007627 |
| FPG3 | USA | Coho salmon | 1947 | Complete | CP007207 |
| FPG101 | USA | Rainbow trout | 2008 | Complete | CP007206 |
