# Supplementary material for: Comparative Genome Analysis Provides Insights into the Pathogenicity of Flavobacterium psychrophilum
Source: PLoS One. 2016 Apr 12;11(4):e0152515. doi: 10.1371/journal.pone.0152515 (PMC4829187; doi:10.1371/journal.pone.0152515)
Supplement: S2 Table — (DOCX) [file pone.0152515.s003.docx]

**Characterization of Chilean *F. psychrophilum* isolates**

Contigs unligned with the reference genome *F. psychrophilum* strain JIP02/86. These contigs were localized at the end of the bacteria chromosome

Table 2S. Genome characteristics of Chilean *F. psychrophilum* isolates.

| Isolate | Number of contigs not aligned with *F. psychrophilum* JIP02/86 | Sum of the length of contigs | Percent of ambiguous bases in the genome (%) |
| --- | --- | --- | --- |
| MH1 | 1 | 3211 | 0,006 |
| PG2 | - | 0 | 0,001 |
| VQ50 | 1 | 2567 | 0,005 |
| 3 | - | 0 | 0,007 |
| 5 | - | 0 | 0,008 |
