# Supplementary material for: Comparative Genome Analysis Provides Insights into the Pathogenicity of Flavobacterium psychrophilum
Source: PLoS One. 2016 Apr 12;11(4):e0152515. doi: 10.1371/journal.pone.0152515 (PMC4829187; doi:10.1371/journal.pone.0152515)
Supplement: S3 Table — (DOCX) [file pone.0152515.s004.docx]

**Distribution of putative virulence factors in *F. psychrophilum* isolates**

Presence of absence of previously identified virulence factors in the *F. psychrophilum* isolates JIP02/86 and FPG3 were studied in all our genome sequences. The table shows an equally distribution of these virulence factors.

Table 3S. Distribution of putative virulence factors across *F. psychrophilum* isolates

|  |  |  |  |  |  | **Strains** |  |  |  |  |  |  |
| --- | --- | --- | --- | --- | --- | --- | --- | --- | --- | --- | --- | --- |
| Locus tag and gene category | Product gene | 950106-1/1 | JIPO2/86 | CSF 259-93 | FPG3 | FPG101 | MH1 | PG2 | VQ50 | 3 | 4 | 5 |
| **proteases**^a^ |  |  |  |  |  |  |  |  |  |  |  |  |
| FP0081 | Putative Zn metalloprotease precursor | + | + | + | + | + | + | + | + | + | + | + |
| FP0082 | Metalloprotease precursor | + | + | + | + | + | + | + | + | + | + | + |
| FP0086 | Metalloprotease PepO precursor | + | + | + | + | + | + | + | + | + | + | + |
| FP0231 | Metalloprotease Fpp1 precursor | + | + | + | + | + | + | + | + | + | + | + |
| FP0232 | Metalloprotease Fpp2 precursor | + | + | + | + | + | + | + | + | + | + | + |
| FP0280 | Putative fungalysin metalloprotease precursor | + | + | + | + | + | + | + | + | + | + | + |
| FP0281 | Putative fungalysin metalloprotease precursor | + | + | + | + | + | + | + | + | + | + | + |
| FP1619 | Putative cytophagalysin metalloprotease precursor | + | + | + | + | + | + | + | + | + | + | + |
| FP1763 | Putative subtilisin family serine endopeptidase precursor | + | + | + | + | + | + | + | + | + | + | + |
| FP2364 | Putative membrane-aasociated Zn metalloprotease precursor | + | + | + | + | + | + | + | + | + | + | + |
| FP2369 | Putative subtilisin family serine endopeptidase precursor | + | + | + | + | + | + | + | + | + | + | + |
| **adhesion**^a^ |  |  |  |  |  |  |  |  |  |  |  |  |
| FP0166-FP0180^c^ | LRR (leucine rich repeat) tandem | + | + | + | + | + | + | + | + | + | + | + |
| FP2413 | Putative adhesin | + | + | + | + | + | + | + | + | + | + | + |
| FP1830 | Putative adhesin | + | + | + | + | + | + | + | + | + | + | + |
| FP0016 | Putative adhesin | + | + | + | + | + | + | + | + | + | + | + |
| FP0595 | Putative adhesin | + | + | + | + | + | + | + | + | + | + | + |
| FP0006 | Putative adhesin | + | + | + | + | + | + | + | + | + | + | + |
| FP1655 | Putative adhesin | + | + | + | + | + | + | + | + | + | + | + |
| FP1499 | Putative adhesin | + | + | + | + | + | + | + | + | + | + | + |
| FP1661 | Putative adhesin | + | + | + | + | + | + | + | + | + | + | + |
| FP1171 | Putative adhesin | + | + | + | + | + | + | + | + | + | + | + |
| FP1959 | Putative adhesin | + | + | + | + | + | + | + | + | + | + | + |
| FP2244 | Putative adhesin | + | + | + | + | + | + | + | + | + | + | + |
| FP0616 | Putative adhesin | + | + | + | + | + | + | + | + | + | + | + |
| **Transport**^b^ |  |  |  |  |  |  |  |  |  |  |  |  |
| FPG3_00490 | Iron transport protein FeoA | - | + | + | + | + | + | + | + | + | + | + |
| FPG3_00495 | Iron transport protein FeoB | + | + | + | + | + | + | + | + | + | + | + |
| FPG3_06485 | Putative hemolysin D transporter | - | - | + | - | + | - | - | - | - | + | - |
| FPG3_10400 | Putative hemolysin D transmembrane transporter | + | + | + | + | + | + | + | + | + | + | + |
| **Motility**^a^ |  |  |  |  |  |  |  |  |  |  |  |  |
| FP0252 | Gliding motility GldA | + | + | + | + | + | + | + | + | + | + | + |
| FP2069 | Gliding motility GldB | + | + | + | + | + | + | + | + | + | + | + |
| FP2068 | Gliding motility GldC | + | + | + | + | + | + | + | + | + | + | + |
| FP1663 | Gliding motility GldD | + | + | + | + | + | + | + | + | + | + | + |
| FP1089 | Gliding motility GldF | + | + | + | + | + | + | + | + | + | + | + |
| FP1090 | Gliding motilityGgldG | + | + | + | + | + | + | + | + | + | + | + |
| FP0024 | Gliding motility GldH | + | + | + | + | + | + | + | + | + | + | + |
| FP1892 | Gliding motility GldI | + | + | + | + | + | + | + | + | + | + | + |
| FP1389 | Gliding motility GldJ | + | + | + | + | + | + | + | + | + | + | + |
| **PorSS secretion system**^a^ |  |  |  |  |  |  |  |  |  |  |  |  |
| FP1973 | PorSS component, GldK | + | + | + | + | + | + | + | + | + | + | + |
| FP1972 | PorSS component, GldL | + | + | + | + | + | + | + | + | + | + | + |
| FP1971 | PorSS component, GldM | + | + | + | + | + | + | + | + | + | + | + |
| FP1970 | PorSS component, GldN | + | + | + | + | + | + | + | + | + | + | + |
| FP2121 | PorSS component, SprA | + | + | + | + | + | + | + | + | + | + | + |
| FP2467 | PorSS component, SprE | + | + | + | + | + | + | + | + | + | + | + |
| FP0326 | PorSS component, SprT | + | + | + | + | + | + | + | + | + | + | + |

“+” presence of the gene

“-“ absence of the gene

a locus tags were based from *F. psychrophilum* isolate JIP02/86

b locus tags were based from *F. psychrophilum* isolate FPG3

c Number of Leucine reach repeat proteins varied among *F. psychophilum* isolates
