# Supplementary material for: Comparative Genome Analysis Provides Insights into the Pathogenicity of Flavobacterium psychrophilum
Source: PLoS One. 2016 Apr 12;11(4):e0152515. doi: 10.1371/journal.pone.0152515 (PMC4829187; doi:10.1371/journal.pone.0152515)
Supplement: S4 Table — (DOCX) [file pone.0152515.s005.docx]

**Genomic Islands identified using the bioinformatics tool PAI finder**

Genomic islands (GIs) were identified in the *F. psychrophilum* isolates 950106-1/1 and CSF 259-93. General characteristics of these specific regions are showed in the table 4S.

Table 4S. Unique genome regions identified in *F. psychrophilum* isolates using PAI finder**.**

| **Region number** | **Position genome** | **Strain** | **Predicted key functions** | **GC%** | **Size (bp)** | **Gene number** |
| --- | --- | --- | --- | --- | --- | --- |
| 1 | 2311523-2377359 | 950106-1/1 | Transposase/transport/toxin/modification-restriction/unknowns | 28.5 | 25836 | 20 |
| 2 | 949811-957339 | CSF 259-93 | Transposases/tetracycline resistance/unknowns | 31.4 | 7528 | 6 |
| 3 | 631631-678513 | CSF 259-93 | Transposase/Integrase/virulence factors/modification-restriction systems/unknowns | 29.8 | 46882 | 35 |
