# Supplementary material for: Comparative Genome Analysis Provides Insights into the Pathogenicity of Flavobacterium psychrophilum
Source: PLoS One. 2016 Apr 12;11(4):e0152515. doi: 10.1371/journal.pone.0152515 (PMC4829187; doi:10.1371/journal.pone.0152515)
Supplement: S5 Table — (DOCX) [file pone.0152515.s006.docx]

**Unique regions found in *F. psychrophilum* isolates using the bainformative program MAUVE**

Accessory regions were found in *F. pscyrhophilum* isolates 950106-1/1, CSF 259-93, 4 and FPG3 by the genome comparison using MAUVE. For the *F. psychrophilum* strain 4 contigs were concatenated in a single sequence. The position in the genome, key functions of ORFs, size and GC% are showed in the table 5S.

Table 5S. Unique genome regions identified in *F. psychrophilum* isolates using MAUVE program**.**

| **Region number** | **Position** | **Strain** | **Predicted key functions** | **GC%** | **Size (bp)** | **Gene number** |
| --- | --- | --- | --- | --- | --- | --- |
| 1 | 2114851-2122666 | 950106-1/1 | Helicase/unknowns | 33.4 | 7816 | 4 |
| 2 | 1308976-1311969 | 950106-1/1 | Unknowns | 24.7 | 3004 | 4 |
| 3 | 73421-75579 | CSF 259-93 | Unknown/ abortive infection system | 27 | 2159 | 2 |
| 4 | 123654-129655 | CSF 259-93^a^ | DNA polymerase IV/unknowns | 33.8 | 6002 | 6 |
| 5 | 187091-193502 | CSF 259-93 | DNA binding protein/unknowns | 30.5 | 6412 | 7 |
| 6 | 521129-527207 | CSF 259-93^a^ | Transport/unknown/transcriptional regulator | 31.5 | 6079 | 5 |
| 7 | 2056618-2058908 | CSF 259-93 | Hydrolase/unknowns | 47.3 | 2291 | 4 |
| 8 | 2424592-2428318 | CSF 259-93 | Unknowns | 31.0 | 3727 | 4 |
| 9 | 113456-133422 | CSF 259-93 | Tranposases/hydrolase/trasporter/unknowns | 29.8 | 19966 | 21 |
| 10 | 597820-607656 | CSF 259-93 | Transposases/unknowns | 31.9 | 9837 | 12 |
| 11 | 329410-335029 | 4 | dGTPase/unknown | 32.1 | 5572 | 5 |
| 12 | 2371063-2379407 | 4 | Tranposases/hydrolase/unknowns | 38.7 | 8293 | 12 |
| 13 | 18672-25123 | 4 | Modification/unknowns/synthesis/transporter/ | 31.9 | 6452 | 7 |
| 14 | 243012-247090 | 4 | Unknowns/protease | 26.2 | 4079 | 6 |
| 15 | 310558-16320782 | 4 | Recombinase/unknowns/transcriptinal regulator/repressor/reductase/endonuclease | 27.9 | 10225 | 15 |
| 16 | 801793-805763 | 4 | Unknowns | 24.9 | 3971 | 3 |
| 17 | 1022035-1025413 | 4 | Unknown/modification-restriction | 29.4 | 3379 | 3 |
| 18 | 1526247-1532856 | 4 | Unknowns/modification-restriction/DNA-binding | 29.3 | 6610 | 5 |
| 19 | 1546100-1553525 | 4 | Helicase/unknowns | 34.7 | 7426 | 3 |
| 20 | 1636722-1642639 | 4 | Unknowns | 32.1 | 5918 | 6 |
| 21 | 1808192-1818715 | 4 | Unknowns/peptidase/transporters | 28.4 | 10524 | 6 |
| 22 | 1793634-1796944 | 4 | Unknowns | 27.7 | 3311 | 4 |
| 23 | 1864394-1869951 | 4 | Unknowns/hemolysin | 27.1 | 5558 | 4 |
| 24 | 1933027-1936961 | 4 | Endonuclease/unknown/modification | 26.7 | 3816 | 3 |
| 25 | 2400960-2410173 | 4 | Unknowns/transcriptional regulator/integrases | 32.3 | 9214 | 8 |
| 26 | 2433612-2442002 | 4 | Unknowns/integrases | 32.7 | 8742 | 13 |
| 27 | 2445114-2458784 | 4 | Tranposase/unknowns/receptor/DNA metabolism | 35.3 | 13671 | 11 |
| 28 | 2461649-2473996 | 4 | Unknowns/amidase | 35.1 | 12348 | 14 |
| 29 | 2473997-2486702 | 4 | Unknowns/peptidase | 36.6 | 12706 | 18 |
| 30 | 2495602-2505070 | 4 | Unknown/multidrug resistance/transporters/heat-shock/ | 30.4 | 9469 | 7 |
| 31 | 2505071-2509877 | 4 | Transcriptional regulator/unknowns | 31.2 | 4807 | 4 |
| 32 | 2509878-2517213 | 4 | Unknowns/DNA-binding | 30.1 | 7336 | 7 |
| 33 | 2535350-2538878 | 4 | Unknowns | 31.8 | 3529 | 3 |
| 34 | 2538879-2541756 | 4 | Unknowns | 30.7 | 2878 | 3 |
| 35 | 2541757-2545327 | 4 | DNA metabolism/unknowns/transcriptional regulator/transposase | 32.1 | 3571 | 5 |
| 36 | 2545328-2552362 | 4 | Unknowns | 31.2 | 7035 | 7 |
| 37 | 2552363-2559458 | 4 | Unknowns/modification-restriction | 30.6 | 7096 | 5 |
| 38 | 2559459-2564486 | 4 | Unknowns | 31.1 | 5028 | 6 |
| 39 | 2564487-2567389 | 4 | Unknowns | 33.2 | 2912 | 4 |
| 40 | 2572044-2576713 | 4 | Unknowns/lipoprotein | 24.2 | 4670 | 6 |
| 41 | 2576714-2582158 | 4 | Unknowns | 30.6 | 5445 | 6 |
| 42 | 2582159-2587099 | 4 | Unknowns | 31.6 | 4941 | 3 |
| 43 | 2587100-2591961 | 4 | Unknown/modification/metabolism | 31.8 | 4862 | 3 |
| 44 | 2591962-2596525 | 4 | Unknowns | 30.0 | 4563 | 7 |
| 45 | 2596526-2599760 | 4 | Unknowns | 32.9 | 3235 | 4 |
| 46 | 2599761-2602828 | 4 | Unknowns | 32.4 | 3068 | 6 |
| 47 | 2602829-2606101 | 4 | Unknowns | 35.2 | 3273 | 5 |
| 48 | 2607824-2611537 | 4 | Unknowns/phage-related | 36.9 | 3714 | 5 |
| 49 | 2637138-2640105 | 4 | Unknowns | 31.9 | 2953 | 3 |
| 50 | 2629290-2632344 | 4 | Unknowns | 33.8 | 3052 | 3 |
| 51 | 2646751-2650241 | 4 | Unknowns | 29.8 | 3491 | 5 |
| 52 | 2650242-2652468 | 4 | Unknowns | 29.9 | 2727 | 3 |
| 53 | 2658389-2661065 | 4 | Peptidase/unknown/Transcriptional regulator | 27.4 | 2677 | 3 |
| 54 | 2262568-2664951 | 4 | Unknowns | 30.3 | 2368 | 4 |
| 55 | 2667708-2669985 | 4 | Unknowns | 28.3 | 2278 | 3 |
| 56 | 2678825-2680444 | 4 | Unknowns | 29.5 | 1620 | 3 |
| 57 | 219789-221530 | FPG3 | Unknown/cell surface protein | 33.7 | 1742 | 2 |
| 58 | 320565-330050 | FPG3 | Unknown/ transcriptional regulator | 24.9 | 9486 | 8 |
| 59 | 1717952-1722504 | FPG3 | Modification/unknowns | 33.9 | 4533 | 4 |
| 60 | 1073771-1082125 | FPG3 | Modification/unknowns | 29.1 | 8355 | 6 |
| 61 | 1412864-1417220 | FPG3 | Unknowns | 25.7 | 4357 | 4 |
| 62 | 743864-746560 | FPG3 | DNA-binding/unknowns | 30.9 | 2697 | 4 |
| 63 | 676034-689433 | FPG3 | Metabolism/unknowns | 30.3 | 13400 | 12 |
| 64 | 609109-613118 | FPG3 | DNA metabolism/ATPase/unknowns | 30.3 | 4010 | 6 |
| 65 | 594039-597563 | FPG3 | Modification/unknowns | 29.9 | 3525 | 4 |

^a^Region also found *F. psychrophilum* strain FPG101
