# Supplementary material for: Comparative Genome Analysis Provides Insights into the Pathogenicity of Flavobacterium psychrophilum
Source: PLoS One. 2016 Apr 12;11(4):e0152515. doi: 10.1371/journal.pone.0152515 (PMC4829187; doi:10.1371/journal.pone.0152515)
Supplement: S6 Table — (DOCX) [file pone.0152515.s007.docx]

**Target genes on bacteriophage 6H by CRISPR array.**

Two CRISPR arrays were identified among all the *F. spychrophilum* isolates. Spacers matched on prophage 6H, which has been previously characterized. The numbers of spacers, gene target and putative functions are shown in the table 6S.

Table 6S. Target genes on bacteriophage 6H by CRISPR spacers

| **CRISPR array** | **Spacer number (sequence)** | **Gene target (accession number)** | **Function** |
| --- | --- | --- | --- |
| CRISPR 1 | 5 (GAATGCGTGCGCTGTATGTATTTCGTTTT) | Hypothetical protein (YP_008320416.1) | Unknown |
|  | 19 (AGTGCTACCTTCGATAATTAGCGCATTAAT) | Hypothetical protein (YP_008320457.1) | Unknown |
| CRISPR 2 | 1 (CTAAAGAGGCTGTTTTATCATCTGTAAATG) | Hypothetical protein (YP_008320473.1) | Unknown |
|  | 2 (GTAGGTGAAGGCGCTGGCGATATGCCAGA) | Putative phage tail protein (YP_008320418.1) | Structural |
|  | 3 (TGCCTTAATGGCTTCAATGTCATTAAATCC) | Putative phage tail protein (YP_008320418.1) | Structural |
|  | 5 (CAAATTTTCGACGAAGTTCGTAAAATTCG) | Hypothetical protein (YP_008320432.1) | Unknown |
|  | 19 (AAAAATCGCTCTTTCTATATTTGAATTTT) | Hypothetical protein (YP_008320465.1) | Unknown |
|  | 34 (CTGTAGATGAAAACGGAAGTTTCCCAATA) | Hypothetical protein (YP_008320472.1) | Unknown |
|  | 45 (CAGCCACAGAACGAACACTTTCCTCACCGA) | Hypothetical protein (YP_008320458.1) | Unknown |
|  | 52 (ATGTTTCGGAGGTAAAATCTAAAATTGTAA) | Hypothetical protein (YP_008320446.1) | Unknown |
|  | 53 (CGACACGTTCGGGATTAGATAGCGTAATA) | Hypothetical protein (YP_008320432.1) | Unknown |
|  | 54 (ACATTGAGCGAAACGACTGTTATGCCTTGT) | Hypothetical protein (YP_008320467.1) | Unknown |
